# International matches elicit stable mechanical workload in high-level female ice hockey

**DOI:** 10.5114/biolsport.2022.109455

**Published:** 2021-10-25

**Authors:** Jérôme Perez, Franck Brocherie, Antoine Couturier, Gaël Guilhem

**Affiliations:** 1 French Institute of Sport (INSEP), Laboratory Sport, Expertise and Performance (EA 7370), Paris, France; 2 French Ice Hockey Federation, Cergy, France

**Keywords:** Mechanical demand, Accelerometry, Congested fixture period, Workload monitoring, Skating

## Abstract

This study aimed to quantify in- and between-match characteristics and mechanical workload variations elicited by a congested schedule in high-level female ice hockey. Six players were monitored during four international pre-season exhibition matches against the same opponent. Two different methods (Player Load and Accel’Rate) were used to assess specific mechanical workload. Number of shifts and effective playing time per shift were significantly higher for period 2 (p = 0.03 for both). Mechanical workload intensity (i.e., relative and peak workload) showed a significant (p ≤ 0.05) decrease from period 1 to period 2 and period 3 (*moderate*-to-*large* Cohen’s *d*). All workload variables remained stable between matches (p > 0.25). Team variability showed *good*-to-*moderate* CVs (< 10%) for all variables for in- and between-match variability. Accumulated workload computed with the Player Load method was threefold higher compared to the Accel’Rate method (+ 87.8% mean difference; *large* Cohen’s *d*). These findings demonstrate that high-level female ice hockey-specific mechanical workload declines with reduced high-intensity output across periods, while it remains stable between matches against standardized opposition. This study strongly suggests that the present workload metrics could be used to determine the mechanical demand elicited by matches played against various opponents in real game conditions.

## INTRODUCTION

Ice hockey is characterized by short and high-intensity efforts interspersed with long recovery periods on the bench. Time-motion analyses (TMA) confirmed this observation as reflected by a low work-to-rest ratio [1]. However, such data are (i) scarce in ice hockey [1–5] and (ii) qualitative in nature and in turn unable to reflect playing intensity [6].

The development of wearable technology such as inertial measurement units (IMUs) has allowed for more detailed and objective quantification of sport-specific movement demands [i.e., using Player Load (PL)] [7–9]. This measure has been widely used in different team sports during competition or training to quantify players’ mechanical workload [10–13]. However, its application during ice hockey tasks remains specific (i.e., gliding sport) due to the movement demands associated with skating stride [14–16]. Previous studies [15, 16] have shown that mechanical workload drops by ˜8% and ˜13% from the first to the second and third period, respectively. This decline has also been reported in video-based TMA studies in men’s ice hockey and might be attributed to the accumulation of both progressive and transient fatigue experienced throughout the match [1, 5, 6].

To date, most of the studies have investigated the locomotor demand using objective measures of mechanical workload across the course of a match [3, 14, 16] or throughout an entire season [15]. However, major competitions (World Championship, Olympic Game or national championship play-off) require players to perform successive matches over 7–10 days with no more than 48 h in between. Only one study [6] has investigated mechanical workload using a local positioning system (LPS) during an Under-20 Men’s World championship tournament but did not examine the influence of congested match schedule on workload metrics. For instance, contextual factors (e.g., opponent level, match period or consecutive matches) should be considered because they might influence workload metrics output [17]. Therefore, it remains challenging to reliably detect mechanical workload (mainly PL data) variations induced by successive matches played over a congested schedule.

Standardized data processing methods that allow accurate assessment of sport-specific activity are recommended. Indeed, excluding stoppage and benching from match analysis [1] may limit the potential underestimation of the most intense periods [18] due to flying and unlimited substitutions as observed in basketball [19]. In addition, an alternative computation method of mechanical workload indexes could be implemented to overcome the fictitious rate of changes in acceleration components generated by the device orientation changes [20].

Therefore, this study aimed to quantify in- and between-match mechanical workload variations elicited by successive international matches against the same opponent (i.e., standardized opposition) in high-level women ice hockey players using IMU-derived measures. We hypothesized that a reduction of the workload will appear across periods and across matches due to fatigue accumulation.

## MATERIALS AND METHODS

### Participants

Based on an international classification [21], ten high-level female ice hockey players belonging to the French national team were monitored over four international exhibition matches with official rules against a similar level national team (international ranking: 10^th^ vs. 5^th^, respectively, at the International Ice Hockey Federation’s world ranking at the time of the experiment). Due to players’ availability (i.e., coaching strategies or injury), only six players (age 22.5 ± 4.1 years, height 165.8 ± 4.2 cm, weight 67.3 ± 6.7 kg) participated in all four matches (the inclusion criterion) and were included in the study. All the players belonging to the first three units (3 players in the first unit, 2 players in the second unit and 1 player in the third unit) of the team line-up participated in all matches. They received a clear explanation of the study before providing written consent to participate in conformity with the Declaration of Helsinki.

### Design

An observational design was conducted to compare match characteristics (i.e., number of shifts, effective playing time per shift, per period and per match) and mechanical workload of high-level female ice hockey players during four international pre-season exhibition matches against the same opponent. Matches were played in the same ice rink arena (e.g., 60 × 30 m) within a 5-day period, mimicking the common competition schedule during an international championship with an average resting time of 20 ± 3 h between matches (Table 1).

**TABLE 1:** Team schedule throughout the experimentation.

	Tuesday	Wednesday	Thursday	Friday	Saturday	Sunday
**Morning**	10:30 Training	11:15 Morning skate	11:15 Morning skate	OFF	11:15 Morning skate	OFF
	Duration: 1 h	Duration: 30 min	Duration: 30 min		Duration: 30 min	

**Afternoon**	OFF	19:00 Match 1	19:00 Match 2	15:30 Training	19:00 Match 3	14:00 Match 4
		Score: 0–3	Score: 1–2		Score: 3–2	Score: 0–1
		Duration: 15 min on-ice warm-up + 3 × 20 min	Duration: 15 min on-ice warm-up + 3 × 20 min	Duration: 1 h	Duration: 15 min on-ice warm-up + 3 × 20 min	Duration: 15 min on-ice warm-up + 3 × 20 min

### Methodology

### Match characteristics

Each match was recorded using a video camera (HDR-CX405 full-HD, Sony, Germany, 50 Hz sampling frequency) with a fixed optical system making it possible to record all players’ (i.e. a total of 24 observations over the 6 matches) activity including benching.

### Mechanical workload processing

During each match, players wore the same trunk-mounted IMU devices (MinimaxX S4, Catapult Innovations, Melbourne, Australia) that include a 100 Hz in-built tri-axial accelerometer, gyroscope and magnetometer. Each unit was positioned in the upper back (i.e., between the scapulae) of each player using a standard vest and were turned on 10 min before each match. Tri-axial accelerometer raw data were exported and synchronized with the video footage using Origin Pro software (Origin 2020, OriginLab Corporation, Northampton, MA). On-ice stoppage, inter-period, and benching times were excluded from the analysis. IMU-derived measures appeared to be reliable and accurate to detect collisions during ice-hockey matches [22]. As spikes in accelerometer data could occur during locomotor movements, video analysis of spikes was used to define collisions as (i) board contact with no body-checking; (ii) board contact with body-checking; (iii) open ice body-checking; and (iv) player fall [22]. Each action meeting the abovementioned criteria that resulted in an instantaneous PL ≥ 2 a.u. was excluded from the analysis.

Mechanical workload was computed using: (i) Player Load (PL), often used during indoor activity [8, 9] and more specifically during ice hockey tasks showing a moderate-to-large test-retest reliability [23], and (ii) Accel’Rate (AR), an alternative computation method less sensitive to sensor rotations [20].

AR and PL were expressed as accumulated mechanical workload (a.u.). PL and AR were normalized to the effective playing time (PL · min^-1^ and AR · min^-1^, a.u. min^-1^).

Previous studies recommend using rolling averages to better describe peak mechanical workload (i.e., peak PL and peak AR, a.u.) [19, 24–26]. The duration of the overlap between the successive windows was set at 20% [25] of effective playing time of all players’ and matches’ shifts. Video footage was used to determine the mean shift effective playing time over all players and matches. All shifts (n = 493) were analysed and effective playing time per shift excluding on-ice stoppage was 45.4 ± 26.1 s. Hence, the duration of the overlap between the successive windows was 9.0 s. All shifts with an effective playing time < 9 s were excluded from the analysis of the peak mechanical workload (n = 10).

### Statistical Analyses

All data were analysed using custom written scripts (Origin 2020, OriginLab Corporation, Northampton, MA) and expressed as mean ± standard deviation (SD). Statistical significance was set at *p* ≤ 0.05. Normality and sphericity were confirmed using the Shapiro-Wilk and Levene’s test, respectively. Differences for match characteristics (effective playing time per shift, period and match) and IMU-derived measures (PL, AR, PL · min^-1^, AR · min^-1^, peak PL and peak AR) covered during each period [period 1 (P1), period 2 (P2) and period 3 (P3)] were analysed using a one-way repeated measures ANOVA (period effect). The same analysis was applied between matches (match effect). Where significant effects were observed, Tukey’s post-hoc test was used to identify specific differences. The relative mean difference and Cohen’s *d* effect size were reported when post-hoc analysis revealed significant differences. Cohen’s *d* was interpreted using the following scale: *trivial* (*d* < 0.2), *small* (*d* = 0.2–0.6), *moderate* (*d* = 0.6–1.2), *large* (*d* = 1.2–2.0) and *very large* (*d* > 2.0) [27]. In- and between-match variability for each player and for the team were examined using the coefficient of variation (CV), rated as *good* (< 5%), *moderate* (5–10%), or *poor* (≥ 10%) [28]. The smallest worthwhile change (SWC) was calculated as 0.2 of the between-participants SD [29].

## RESULTS

### Match characteristics

**FIG. 1:**
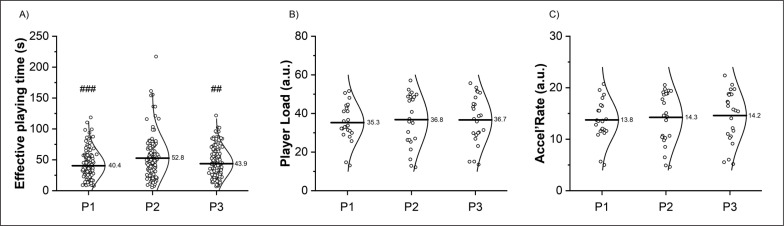
Effective playing time per shift excluding on-ice stoppage (panel A), accumulated workload Player Load (panel B) and Accel’Rate (panel C) by period. P1: period 1; P2: period 2; P3: period 3. ## (*p* ≤ 0.01) and ### (*p* ≤ 0.001) significantly different from P2.

Match characteristics are reported in Tables 2 and 3. A main effect of period was found for the effective playing time per shift (*p* < 0.001). Post-hoc analysis showed a significant *moderate* increase of the effective playing time per shift from P1 to P2 (+ 30.7%; *p* < 0.001, *d* = 0.85) and a significant *moderate* decrease from P2 to P3 (-16.9%; *p* = 0.01, *d* = 0.62) (Figure 1).

**TABLE 2:** Match characteristics and mechanical workload between periods

Variable	Period 1	Period 2	Period 3	Mean (match)	Aggregate (4 matches)
**Number of shifts**	7 ± 1 (6–8)	6 ± 1 (6–7)	7 ± 1 (6–8)	7 ± 2 (6–7)	21 ± 5 (18–23)
**Effective playing time per shift (s)**	40.4 ± 22.0 ### (37.2–43.6)	52.8 ± 32.3 (47.7–57.9)	43.9 ± 22.3 ## (40.7–47.2)	45.4 ± 26.1 (43.1–47.6)	
**Effective playing time (min)**	5.6 ± 0.3 (4.9–6.2)	6.3 ± 0.5 (5.3–7.3)	6.3 ± 0.5 (5.3–7.3)	6.1 ± 2.1 (5.6–6.5)	18.4 ± 5.4 (16.1–20.6)
**PL (a.u.)**	35.32 ± 3.35 (26.71–43.92)	36.79 ± 4.49 (25.25–48.33)	36.70 ± 4.09 (26.20–47.21)	36.27 ± 9.24 (31.68–40.86)	110.89 ± 32.79 (97.05–124.74)
**AR (a.u.)**	13.77 ± 0.78 (12.16–15.37)	14.26 ± 1.04 (12.10–16.42)	14.60 ± 1.02 (12.49–16.71)	14.21 ± 4.62 (13.12–15.30)	43.03 ± 12.49 (37.76–48.31)
**PL** · **min^-1^ (a.u.min^-1^)**	6.61 ± 0.20 (6.11–7.11)	6.07 ± 0.15 ** (5.68–6.46)	6.10 ± 0.08 ** (5.89–6.31)	6.26 ± 0.59 (6.05–6.47)	
**AR** · **min^-1^ (a.u.min^-1^)**	2.55 ± 0.05 (2.42–2.69)	2.32 ± 0.04 *** (2.23–2.42)	2.36 ± 0.02 ** (2.32–2.40)	2.41 ± 0.14 (2.34–2.48)	
**Peak PL rolling average (a.u.)**	0.175 ± 0.008 (0.155–0.194)	0.165 ± 0.003 (0.156–0.174)	0.162 ± 0.007 * (0.145–0.180)	0.167 ± 0.015 (0.160–0.175)	
**Peak AR rolling average (a.u.)**	0.065 ± 0.002 (0.059–0.072)	0.064 ± 0.001 (0.061–0.066)	0.061 ± 0.002 (0.056–0.067)	0.064 ± 0.006 (0.061–0.066)	

Note: Data are displayed as mean ± SD (and 95% confidence interval). PL: Player Load; AR: Accel’Rate.

* (*p* ≤ 0.05),

** (*p* ≤ 0.01) and

*** (*p* ≤ 0.001) significantly different from Period 1.

## (*p* ≤ 0.01) and

### (*p* ≤ 0.001) significantly different from Period 2.

**TABLE 3:** Matches characteristics, mechanical workload and between-match variability

Variables	Matches characteristics and mechanical workload				Between-match variability			

	Mean ± standard deviation (with 95% confidence interval)				Player		Team	

	Match 1	Match 2	Match 3	Match 4	CV (95% CI)	SWC	CV (95% CI)	SWC
**Number of shifts**	20 ± 2 (15–26)	21 ± 2 (17–26)	21 ± 3 (13–29)	20 ± 2 (14–25)	13.1 (8.1–32.0)		3.1 (1.9–7.4)	
**Effective playing time (min)**	18.8 ± 2.0 (13.7–24.0)	18.3 ± 1.5 (14.3–22.2)	19.0 ± 2.9 (11.5–26.5)	17.3 ± 2.7 (10.4–24.2)	19.5 (12.0–47.0)	0.6	4.1 (2.5–9.8)	0.2
**PL (a.u.)**	116.74 ± 13.25 (82.69–150.79)	110.26 ± 10.16 (84.15–136.37)	113.47 ± 16.77 (70.36–156.58)	103.47 ± 15.62 (62.95–143.27)	16.2 (10.0–39.0)	2.93	5.3 (3.1–12.0)	1.17
**AR (a.u.)**	44.05 ± 4.49 (32.52–55.59)	43.54 ± 4.19 (32.76–54.32)	44.58 ± 6.50 (27.86–61.29)	39.96 ± 6.08 (24.32–55.60)	16.0 (10.0–39.0)	1.11	4.9 (3.1–12.0)	0.42
**PL** · **min^-1^ (a.u.min^-1^)**	6.39 ± 0.22 (5.82–6.97)	6.15 ± 0.12 (5.85–6.45)	6.37 ± 0.12 (6.06–6.68)	6.19 ± 0.16 (5.79–6.59)	4.9 (3.1–12.0)	0.06	1.9 (1.2–4.9)	0.02
**AR** · **min^-1^ (a.u.min^-1^)**	2.42 ± 0.07 (2.24–2.59)	2.39 ± 0.06 (2.23–2.55)	2.46 ± 0.05 (2.33–2.59)	2.40 ± 0.04 (2.30–2.50)	4.7 (3.1–12.0)	0.02	1.3 (0.6–2.5)	0.01
**Peak PL (a.u.)**	0.163 ± 0.011 (0.136–0.191)	0.178 ± 0.002 (0.172–0.186)	0.172 ± 0.008 (0.151–0.192)	0.167 ± 0.007 (0.149–0.184)	7.5 (5.0–20.0)	0.003	4.0 (2.5–9.8)	0.001
**Peak AR (a.u.)**	0.065 ± 0.002 (0.060–0.069)	0.063 ± 0.002 (0.058–0.067)	0.063 ± 0.002 (0.057–0.068)	0.061 ± 0.002 (0.057–0.065)	5.0 (3.1–12.0)	0.0006	2.2 (1.2–4.9)	0.0003

Note: PL: Player Load; AR: Accel’Rate; CV: coefficient of variation (with 95% confidence interval); SWC: smallest worthwhile change.

### In-match mechanical workload demands

No effect of period was found for in-match PL and AR (*p* > 0.55; Figure 1 and Table 2). A main effect of period was found for PL · min^-1^ (*p* = 0.004) and for AR · min^-1^ (*p* < 0.001). Post-hoc analysis showed a significant *moderate* decrease of PL · min^-1^ from P1 to P2 (-8.6%; *p* = 0.006, *d* = -1.09) and period 3 (-8.0%; *p* = 0.009, *d* = -1.16) but not between P2 and P3 (+ 0.5%; *p* = 0.97, *d* = 0.11). Similarly, AR · min^-1^ *largely* decreased from P1 to P2 (-9.4%; *p* < 0.001, *d* = -1.44) and P3 (-8.1%; *p* = 0.002, *d* = -1.44) but not between P2 and P3 (+ 1.4%; *p* = 0.69, *d* = 0.50). Considering peak mechanical workload using a rolling average of 9.0 s [25], a significant effect of period was found for peak PL (*p* = 0.05) but not for peak AR (*p* = 0.06). There was a decrease from P1 to P3 (-7.3%; *p* = 0.05, *d* = -0.62 and -6.4%; *p* = 0.05, *d* = -0.72 for peak PL and peak AR, respectively) for both variables. During match 4, workload variables significantly decreased from P1 to P3 (> -7.5%; *p* > 0.37, *d* ranging from -0.23 to -0.21 for PL and AR, > -11.1%; *p* < 0.001, *d* ranging from -1.29 to -1.52 for relative values and > -10.6%; *p* < 0.05, *d* ranging from -0.75 to -1.13 for peak workload).

### Between-match mechanical workload demands

**FIG. 2:**
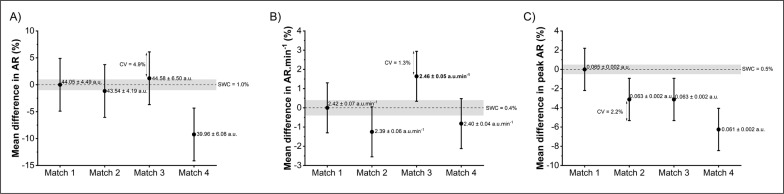
Relative mean difference between matches compared to Match 1 for accumulated mechanical workload Accel’Rate (AR; panel A), relative value of Accel’Rate (AR · min^-1^; panel B) and peak workload using a rolling average of 9 s (peak AR; panel C). Absolute values of each measure are displayed as mean ± standard deviation. Coefficient of variation (CV) represents the between-match variability of each measure and the grey areas, called the smallest worthwhile change (SWC), represent trivial change.

No significant difference was found for accumulated (*p* > 0.69 for PL and AR), relative values (*p* > 0.47 for PL · min^-1^ and AR · min^-1^) and peak mechanical workload (*p* > 0.25 for peak PL and peak AR; Figure 2).

### In- and between-match variability

In-match player CV for the change in IMU-derived measures were considered as *good*-to-*moderate* (CVs ranging 4.5%-9.2%) with the lowest value obtained for the peak mechanical workload and the highest value for the accumulated mechanical workload. Compared to player variability, higher CVs (ranging from 3.2% for the peak mechanical workload to 5.1% for relative mechanical workload) were observed for all the team variables. Mechanical workload was not significantly different between matches, regardless of the considered indicator (Table 3).

### Data processing and computation method

Higher PL and AR were obtained for the match when considering the entire 20-min period (175.68 ± 41.29 a.u. and 68.17 ± 16.74 a.u. for PL and AR, respectively) compared to effective playing time (110.89 ± 32.79 a.u. and 43.03 ± 12.49 a.u. for PL and AR, respectively). Inversely, relative values were higher considering only shifts compared to the entire 20-min period (6.26 ± 0.59 a.u.min^-1^ vs. 1.95 ± 0.36 a.u.min^-1^). PL was three times higher than AR (+ 87.8% mean difference; *d* = 1.21) for the same accumulated workload analysis.

## DISCUSSION

To the authors’ knowledge, this study is the first to quantify in- and between-match characteristics and mechanical workload variations elicited by a congested schedule with standardized opposition in high-level female ice hockey. Reported mechanical workload (i.e., relative and peak workload using rolling averages) significantly decreased across the periods. Inversely, accumulated mechanical workload was stable with low variability (CV < 10%) between matches. From a methodological point of view, mechanical workload quantification using AR consistently showed lower values compared to PL, in line with previous data obtained in running [20].

While the players monitored here performed a higher number of shifts compared to female ice hockey players during Canadian university matches [3] (21 ± 5 vs. 16), effective playing time per shift remained similar (˜45 s). In this context, total and peak AR and PL *moderately* decreased by ˜8.5% and ˜5.5% respectively from P1 to P3. This finding aligns with previous TMA [1, 5] or LPS/IMU technologies [6, 15, 16]. Our results confirm this decline in high-intensity output across periods (Table 2), to a similar extent as in elite male American Hockey League (-4.5% and -6.0% from P1 to P2 and P3, respectively) [15]. Douglas et al. [16] also reported a similar decrease in explosive effort metrics in P2 (-10.0%) and P3 (-8.0%) compared to P1. Using pre- and post-match biopsies, a recent study attributed this decline in explosive performance to pronounced glycolytic loading resulting in a marked decrease of muscle glycogen (-53%) [30].

The present protocol, mimicking a congested schedule similar to a World Championship tournament, should be considered when interpreting the present variations in IMU-derived measures [17]. Contrarily to our hypothesis, the results did not show any changes in workload across matches. Such standardization of match opposition may differ from official tournaments where the team plays against various opponents. Alternatively, the high-level players participating in this study were accustomed to this type of congested schedule during international competitions. In addition, ice hockey is a gliding sport with a relatively low volume of effective playing time compared to the aforementioned sports (18.4 ± 5.4 min). The lower workload and ground impacts may lead to a lower amount of exercise-induced muscle damage compared to stretch-shortening cycle activities such as sprint running or field team sports [5]. This potential reduced traumatic loading may shorten the time required for a sufficient post-match recovery and favours match repetition. It is important to note that while no significant difference was found between matches, the last match (match 4) showed a *small* drop for the accumulated workload (˜-9.0%) and a *moderate* drop for the peak workload (˜-6.0%) compared to the first match. In addition, match 4 was the only match showing a decrease for all variables from P1 to P3. Considering that the decrease in workload does not result from a change in opponent team (i.e. standardized opposition), this decline could reflect a residual fatigue accumulated during the previous matches for either the players monitored or the opponents. While we cannot rule out that similar accumulated fatigue affected the performance of the opponent team, one may assume that these effects may be amplified during an official competition with higher competitiveness and accompanying stress.

Although challenging, standardizing match conditions and contextual factors as much as possible is required to reliably detect variations in mechanical workload. This study is the first investigating the variability of different IMU-derived measures over standardized international match conditions in ice hockey (same opponent, place, team composition), during a congested schedule. Team variability showed *good*-to-*moderate* (< 10%) CVs for all variables for in- and between-match variability. Considering player variability, only accumulated workload showed the highest value with *poor* (> 10%) CVs for between-match variability. These variables are directly linked to match characteristics, especially effective playing time per period or match, which also showed high variability for both in- and between-match values. Specific ice hockey rules (e.g., unlimited substitutions, penalty situations) allowed coaches to make tactical adjustments especially during P3 depending on the match outcome [3, 16].

Previously reported IMU-derived measures in female ice hockey matches considered the entire 20-min period without taking into account the specific nature of the sport (brief, high-intensity bouts interspersed with long recovery periods) [14, 16]. Such data processing may lead to an overestimation of the accumulated mechanical workload and an underestimation of the high-intensity effective playing time. Using the method proposed by Douglas et al. [14], the present data demonstrate lower PL (175.68 ± 41.29 a.u. vs. ˜230 a.u.) and PL · min^-1^ (1.95 ± 0.36 a.u.min^-1^ vs. ˜2.20 a.u.min^-1^) than elite level competitions but similar PL (175.68 ± 41.29 a.u. vs. 183.0 ± 44.3 a.u) and higher PL · min^-1^ (1.95 ± 0.36 a.u.min^-1^ vs. 1.8 ± 0.4 a.u.min^-1^) compared to sub-elite level competitions [31]. The differences from elite level competitions could reflect the differences in performance level of the players monitored (i.e., 10^th^ vs. 2^nd^ position in the International Ice Hockey Federation’s world ranking). However, regarding the highest intensity completed by the players monitored in the present study compared to sub-elite players, it seems that the international level could be considered as a higher level compared to the Canadian collegiate level [31].

Ice hockey elicits numerous collisions, changes in directions, cutting manoeuvres and complex motor tasks performed at extremes joint amplitudes and high intensity. Such movements substantially modify accelerometer orientation, which may in turn generate a fictitious rate of changes in acceleration components [7]. Using an alternative computation method of mechanical workload (AR) [20], all AR-derived variables were lower compared to PL-derived variables (divided on average by 2.6, -87.8% mean difference), while CV ratings were closer. In line with Hollville et al. [20], these findings indicated that the AR method could be a stable (i.e., less sensitive to sensor rotations) and valid PL metric.

The present study provides important insights for female ice hockey practitioners; however, some limitations should be considered. First, the small sample size precludes any general conclusions and does not allow us to further differentiate specific player positions that may have an impact on the match characteristics and IMU-derived measures (i.e., statistical power ranged from 0.17 for relative workload to 0.87 for accumulated workload). However, we accumulated repeated observations over successive matches to provide a more reflective overview of ice hockey specific mechanical workload [3, 14, 16]. Second, according to Lacome et al. [25], the overlap between the successive windows for the moving average was set at 20% of the effective playing time per shift. Further studies should investigate the effect of different processing periods on workload, given its crucial implication to implement appropriate training prescription based on drill duration [19].

### Practical applications

Our results could provide mechanical workload reference values in a real-world setting for other female high-level ice hockey teams facing a congested schedule (i.e., major ice hockey competitions). Monitoring the mechanical workload using wearable technology in this specific context may allow coaching staff to optimize each individual player performance. Players with high mechanical workload (i.e., long effective playing time) leading to a decrease in high-intensity output may be preserved thanks to coaching strategies or oriented towards recovery strategies between matches. Conversely, players exposed to lower mechanical workload may benefit from a quick, high-intensity, additional post-match workout (i.e., micro-dosing; [32]) or during a competition a day off to keep them in good shape for the rest of the competition. Finally, it appears that recovery strategies should be prioritized with competition progress to limit the effect of accumulated fatigue, which could partly explain the decrease in all mechanical workload output.

## CONCLUSIONS

In conclusion, while high-intensity output moderately-to-largely declined across periods (i.e., ˜-8.5% for relative workload and ˜-6.5% for peak workload), the present study shows that workload metrics remain significantly stable (all *p* > 0.25) in high-level female ice hockey matches played against standardized opposition. The effect of accumulated fatigue seems to induce a reduction of mechanical workload during the fourth match. Such information could be helpful to coaches to prepare physical fitness and tactics according to the competitive schedule. In addition, the monitoring of such metrics may contribute to better individualisation for detecting a possible fatigue effect or a lower mechanical workload due to match/coaching scenario. A more position-specific analysis is warranted to better identify congested workload demand and to enhance the training process.

## References

[1] Brocherie F, Girard O, Millet GP. Updated analysis of changes in locomotor activities across periods in an international ice hockey game. Biol Sport. 2018;35(3):261–7. 30449944 10.5114/biolsport.2018.77826 PMC6224850

[2] Bracko M, Fellingham G, Hall L, Fisher A, Cryer W. Performance skating characteristics of professional ice hockey forwards. Res Sports Med. 1998;8(3):251–63.

[3] Jackson J, Snydmiller GD, Game AB, Gervais P, Bell GJ. Movement characteristics and heart rate profiles displayed by female university ice hockey players. Int J Kinesiol Sport Sci. 2016;4(1):43–54.

[4] Jackson J, Snydmiller GD, Game AB, Gervais P, Bell GJ. Investigation of positional differences in fitness of male university ice hockey players and the frequency, time spent and heart rate of movement patterns during competition. Int J Kinesiol Sport Sci. 2017;5(3):6–15.

[5] Lignell E, Fransson D, Krustrup P, Mohr M. Analysis of high-intensity skating in top-class ice hockey match-play in relation to training status and muscle damage. J Strength Cond Res. 2018;32(5):1303–10. 28557852 10.1519/JSC.0000000000001999

[6] Douglas A, Kennedy C. Tracking in-match movement demands using local positioning system in world-class men’s ice hockey. J Strength Cond Res. 2020;34(3):639–46. 31855927 10.1519/JSC.0000000000003414

[7] Roell M, Roecker K, Gehring D, Mahler H, Gollhofer A. Player monitoring in indoor team sports: Concurrent validity of inertial measurement units to quantify average and peak acceleration values. Front Physiol. 2018;9(141). 10.3389/fphys.2018.00141 PMC5835232 29535641

[8] Luteberget LS, Holme BR, Spencer M. Reliability of wearable inertial measurement units to measure physical activity in team handball. Int J Sports Physiol Perform. 2018;13(4):467–73. 28872371 10.1123/ijspp.2017-0036

[9] Boyd LJ, Ball K, Aughey RJ. The reliability of MinimaxX accelerometers for measuring physical activity in Australian football. Int J Sports Physiol Perform. 2011;6(3):311–21. 21911857 10.1123/ijspp.6.3.311

[10] Wik EH, Luteberget LS, Spencer M. Activity profiles in international women’s team handball using playerLoad. Int J Sports Physiol Perform. 2017;12(7):934–42. 27967272 10.1123/ijspp.2015-0732

[11] Luteberget LS, Trollerud HP, Spencer M. Physical demands of game-based training drills in women’s team handball. J Sports Sci. 2018;36(5):592–8. 28508705 10.1080/02640414.2017.1325964

[12] Fox J, Stanton R, Scanlan A. A comparison of training and competition demands in semiprofessional male basketball players. Res Q Exerc Sport. 2018;89(1):103–11. 29334021 10.1080/02701367.2017.1410693

[13] Young CM, Gastin PB, Sanders N, Mackey L, Dwyer DB. Player load in elite netball: Match, training, and positional comparisons. Int J Sports Physiol Perform. 2016;11(8):1074–9. 27001768 10.1123/ijspp.2015-0156

[14] Douglas A, Rotondi MA, Baker J, Jamnik VK, Macpherson AK. On-ice physical demands of world-class women’s ice hockey: From training to competition. Int J Sports Physiol Perform. 2019;14(9):1227–32. 10.1123/ijspp.2018-0571 30859859

[15] Allard P, Martinez R, Deguire S, Tremblay J. In-season session training load relative to match load in professional ice hockey. J Strength Cond Res. 2020;Jan 28 (Epub ahead of print). 10.1519/JSC.0000000000003490 31996615

[16] Douglas A, Johnston K, Baker J, Rotondi MA, Jamnik VK, Macpherson AK. On-ice measures of external load in relation to match outcome in elite female ice hockey. Sports. 2019;7(7):173. 10.3390/sports7070173 PMC6681036 31315209

[17] Dalton-Barron N, Whitehead S, Roe G, Cummins C, Beggs C, Jones B. Time to embrace the complexity when analysing GPS data? A systematic review of contextual factors on match running in rugby league. J Sports Sci. 2020;38(10):1161–80. 32295471 10.1080/02640414.2020.1745446

[18] Whitehead S, Till K, Weaving D, Jones B. The use of microtechnology to quantify the peak match demands of the football codes: A systematic review. Sports Med. 2018;48(11):2549–75. 30088218 10.1007/s40279-018-0965-6 PMC6182461

[19] Fox J, Conte D, Stanton R, McLean B, Scanlan A. The application of accelerometer-derived moving averages to quantify peak demands in basketball: A comparison of sample duration, playing role, and session type. J Strength Cond Res. 2020;Feb 14 (Epub ahead of print). 10.1519/JSC.0000000000003486 34846331

[20] Hollville E, Couturier A, Guilhem G, Rabita G. A novel accelerometry-based metric to improve estimation of whole-body mechanical load. Sensors. 2021;21(10):3398. 10.3390/s21103398 PMC8153011 34068169

[21] Decroix L, De Pauw K, Foster C, Meeusen R. Guidelines to classify female subject groups in sport-science research. Int J Sports Physiol Perform. 2016;11(2):204–13. 26182438 10.1123/ijspp.2015-0153

[22] Pilotti-Riley A, Stojanov D, Sohaib Arif M, McGregor SJ. Video corroboration of player incurred impacts using trunk worn sensors among national ice-hockey team members. PLoS One. 2019;14(6):e0218235. 31233527 10.1371/journal.pone.0218235 PMC6590802

[23] van Iterson EH, Fitzgerald JS, Dietz CC, Snyder EM, Peterson BJ. Reliability of triaxial accelerometry for measuring load in men’s collegiate ice hockey. J Strength Cond Res. 2017;31(5):1305–12. 27548782 10.1519/JSC.0000000000001611

[24] Fereday K, Hills SP, Russell M, Smith J, Cunningham DJ, Shearer D, et al. A comparison of rolling averages versus discrete time epochs for assessing the worst-case scenario locomotor demands of professional soccer match-play. J Sci Med Sport. 2020;23(8):764–9. 31937507 10.1016/j.jsams.2020.01.002

[25] Lacome M, Simpson BM, Cholley Y, Lambert P, Buchheit M. Small-Sided Games in Elite Soccer: Does One Size Fit All? Int J Sports Physiol Perform. 2018;13(5):568–76. 28714774 10.1123/ijspp.2017-0214

[26] Oliva-Lozano JM, Martín-Fuentes I, Fortes V, Muyor JM. Differences in worst-case scenarios calculated by fixed length and rolling average methods in professional soccer match-play. Biol Sport. 2021;38(3):325–31. 34475615 10.5114/biolsport.2021.99706 PMC8329979

[27] Hopkins WG, Marshall SW, Batterham AM, Hanin J. Progressive statistics for studies in sports medicine and exercise science. Med Sci Sports Exerc. 2009;41(1):3–13. 19092709 10.1249/MSS.0b013e31818cb278

[28] Lord C, John Blazevich A, Abbiss CR, Ma’ayah F. A Reduction in match-to-match variability using maximal mean analyses in sub-elite soccer. Int J Sports Med. 2020;41(5):300–5. 31958875 10.1055/a-1073-7851

[29] Hopkins WG. How to interpret changes in an athletic performance test. Sportscience. 2004;8(1):1–7.

[30] Vigh-Larsen JF, Ermidis G, Rago V, Randers MB, Fransson D, Nielsen JL, et al. Muscle Metabolism and Fatigue during Simulated Ice Hockey Match-Play in Elite Players. Med Sci Sports Exerc. 2020;52(10):2162–71. 32496739 10.1249/MSS.0000000000002370

[31] Douglas AS, Rotondi MA, Baker J, Jamnik VK, Macpherson AK. A comparison of on-ice external load measures between subelite and elite female ice hockey players. The Journal of Strength & Conditioning Research. 2020;Aug 12 (Epub ahead of print). 10.1519/JSC.0000000000003771 32796414

[32] Blagrove RC, Hooper DR. Strength training for enhancing performance and reducing injury risk. The Science and Practice of Middle and Long Distance Running. 2021;(Epub ahead of print):207.

